# Homeopathy and Homeopaths under the Calcium Light—Their Claims to Consideration in Medical Legislation

**Published:** 1891-03

**Authors:** 


F. E. DANIEL, M. D., EDITOR AND PUBLISHER.

THIS JOURNAL, by unanimous vote of the members, was made the Official Organ of the Austin District Medical Society.

COLLABORATORS:

*Dr. R. M. Swearingen, Austin.*

*Prof. B. E. Hadra, M. D., Galveston.*

*Prof. Geo. Cupples, M. D., San Antonio.*

*Dr. T. C. Osborn, Cleburne, Texas.*

*Dr. E. J. Doering, Chicago.*

*Dr. E. J. Beall, Fort Worth.*

*Dr. Odo Betz, Germany.*

*Prof. W. B. Rogers, M. D., Memphis.*

*Dr. J. W. McLaughlin, Austin.*

*Dr. T. J. Tyner, Austin.*

*Prof. J. F. Y. Paine, M. D., Galveston.*

*Dr. R. H. L. Bibb, Mexico.*

*Dr. T. J. Bennett, Austin.*

*Dr. Bat Smith, Wharton.*

*Dr. E. Meierhof, New York.*

*L. H. Luce, M. D., West Tisbury, Mass.*

### HOMEOPATHY AND HOMEOPATHS UNDER THE CALCIUM LIGHT.

### Their Claims to Consideration as Factors in Medical Legislation.

THERE ARE NO SECTS in medicine; all the recent homeopathic outcry about the "three schools," and the "different systems of medicine," etc., is the meerest twaddle.

Homeopathy, as defined by DUNGLISON,—the standard authority with all classes of practitioners—is "a fanciful doctrine which maintains that disordered actions in the human body are to be cured by inducing other disordered actions of a like kind, (*similia similibus*,) and this to be accomplished by infinitesimally small doses, often of apparently inert agents; the decillionth part of a grain of charcoal, for example, is an authorized dose. It has also been called 'globulism.' " According to Dr. Bigelow "homeopathy consists in leaving the case to nature, while the patient is amused with nominal and nugatory remedies."

WEBSTER defines it to be "the doctrine or theory of curing diseases with very minute doses of medicine, by producing in the patient affections similar to those of the disease."

WORCESTER says, "Homeopathy is the doctrine of Dr. Hahnemann that diseases are cured by medicines which have the power to cause similar diseases in healthy persons, or the doctrine that *similia similibus curantur*, "like cures like."

THOMAS' MEDICAL DICTIONARY defines it as "a doctrine propounded by Hahnemann, professing to cure diseases by the action of infinitesimal doses of medicines of a quality to excite in the healthy a disease similar to that which is to be cured."

From the above it will be seen that homeopathy is no part of the science of medicine; there is but one "school" of medicine; that founded by Hippocrates, the great science of medicine, which comprehends every and all rational methods of curing disease, and includes biology, physiology, pathology, psychology, chemistry, surgery and hygiene. Its followers and devotees have made the science and themselves illustrious, have advanced civilization, prolonged human life and robbed disease of most of its terrors. They alone have contributed to the vast sum of medical progress and advancement, and have given to suffering humanity *all* the boons with which they are now blessed. Long—the immortal, robbed surgery and obstetrics of their terrors by the discovery of chloroform. By its use the sufferer literally sleeps throughout the most formidable surgical operation. Harvey demonstrated the circulation of the blood and revolutionized physiology. Paré gave the world the ligature,—the next greatest advance in surgical aid. Jenner disarmed smallpox of its terrors by the discovery of vaccination, and saved millions of lives annually. Pasteur has disarmed hydrophobia. Koller gave us local anæsthesia in the discovery of cocaine. Koch, Klebs, Lister and others have revolutionized pathology by their discoveries and demonstrations of the germ causation of disease. In short, the medical firmament is begemmed with stars of the first magnitude.

All "sects" or "schools" of medicine are mere "pathies," nonentities, and represent nothing,—not even a rational idea. And of all the ridiculous and absurd theories ever seriously proposed homeopathy is the most absurd and irrational.

By comparison with the above brilliant record, it can be shown that with all their boast—during the century that has elapsed since Hahnemann brought forth his absurd "system"—so called "of all the vast achievements made in medical science, *not one* important discovery, either in medicine or surgery, can be ascribed to homeopathic origin; there is not one single instance in the world of a single scientist who is a homeopath. There is not one single homeopathic doctor who has more than a local reputation as a scientist. What a proud record! A "sect" in medicine, as alleged, which has existed a hundred years, at a period of the world's history when the stupendous advancements in all departments of science and learning have been such as to put to shame all previous ages, and still this boastful, arrogant "system" has not contributed one iota to this vast addition to scientific knowledge. It has done nothing but degrade the name of medicine, and bring reproach upon the profession."*

With regard to their boast of "sixteen colleges," and of their having "elevated the standard of medical education,"—when it is examined by the calcium light, it is found to be, like a certain green bay tree, "hollow in the butt." It can be shown that it is directly the result of the examining board system, and that they were driven to it. These colleges, along with a number of medical colleges, were made to extend their lecture terms, and make more thorough their course of instruction—or be *ruled out!* In 1881 the Illinois State Board of Health, through their great Secretary Jno. H. Rauch, made a report on medical education, and classified the colleges, saying which should and which should not be recognized as being in "good standing." Other boards adopted the classification, and the published results of examinations of graduates of the different colleges soon made evident the weak schools;—hence, "raise the standard or quit the business" became the alternative.

If the effect on the homeopathic colleges was salutary, it was more so on the medical colleges proper, and there was a great advance all along the lines.

In 1882 only 45 of the 148 medical colleges in the United States and Canada required certain educational qualifications for

---

*Whenever an * is used in this article, reference is made to and quotations from Dr. S. N. Pierce's article in the Medical Standard.

matriculation; now 129, or all but 19 require it. In 1882 the number requiring attendance on three or more courses of lectures, as a condition to graduation, was 22; now it is 85. In 1882-3 only 42 colleges had terms of 6 months or more, at present 111 have; and of the 148 colleges, only 22 adhere to the original low standard, short time, etc.—[*7th Illinois Report on Medical Education.*]

So much, then, for examining boards as factors in "elevating the standard of medical education;" yet these (alleged) homeopaths, while boasting of the elevation of their standard, (wrought by the boards), decry the boards as useless and oppressive.

Speaking of medical colleges, it is not generally known that with the exception of one in Hungary, there is not such a thing as a "homeopathic medical college" outside of these glorious and do-as-you-please United States; and here, while calling themselves homeopaths, these persons practice, or essay to practice, anything that comes handy ! To do this in Germany,—to deviate from the strict homeopathic doctrine that "like cures like" ("the hair of the dog cures the bite"), and infinitesimal doses, is held to be, as it really is, *false pretense*, and is punished as such. (Small wonder then, that homeopathy has become almost totally extinct in Europe, and especially in Germany, the land of its birth !) Such should be the case in America; for it can be proven,—indeed, few deny it,—we challenge those in Texas loudest in their complaint to deny it,—that while posing as homeopaths, getting practice as homeopaths, they do not hesitate to prescribe morphine, calomel, quinine, chloral, etc., in full medicine doses, admitting the meanwhile, as did the President of the forty-strong "Homeopathic State Medical Society" in a recent newspaper publication that the "two systems" are diametrically opposed to each other." The nearest approach to the German ruling that we have heard of, was the decision of a northern court—(not remembered at present, but which can be cited)—that if a homeopath is employed as a homeopath, it is tacily understood that the patient is to be treated homeopathically, and if the homeopath resort to other means or remedies, he cannot enforce the payment of his bill. But, as a general thing, those persons calling themselves homeopaths use the name as a kind of trade-mark, pretty much as the Indian has become the trade-mark of the tobacco dealer; they sail under a homeopathic flag and cry persecution; they declare that the administration of homeopathic medicines is harmless, and claim exemption from examination *as* homeopaths, and *yet*, give morphine and quinine!

Here is the "nigger in the wood-pile," as the homeopathic president of the 40 strong association, elegantly expresses it; we have "flushed him." Homeopaths want the people to believe that they practice only homeopathy, and insist that it is "harmless," and yet reserve the right to, and "on the sly" do administer dangerous drugs in full doses, depart from the fundamental principles of homeopathy and fly to the very opposite! Is this fair? is it rational? is it honorable? When he does so he necessarily abandons *in toto*, the whole theory upon which the absurd practice of homeopathy is founded. What is he then? surely not a homeopath; he is neither fish, flesh nor fowl; nor yet not even "good red herring." They would "steal the livery of (the Hippocratean) heaven "to serve the (Hahnemannian) devil in." This looks, to a man up a tree, singularly like duplicity. Doing this thing, posing as homeopaths and attempting to practice *medicine*, of which they are totally ignorant in the great majority of cases, in the name of consistency and of common sense, upon what do they base their claims to be exempt from examination? Are not surgery, obstetrics, anatomy, chemistry, physiology, hygiene, and all else save therapeutics and practice, taught alike in all schools? And if they essay the latter—the regular practice—where does the homeopathy come in? But the president of the homeopaths above referred to has, in another, and more recent newspaper publication had the—courage, shall we call it? or cheek?—to deny that he had said "the administration of homeopathic remedies is harmless," and asked to be left out of the bill altogether; notwithstanding we "saw it with our eyes, and heard it with our ears," saw it in print in the *Statesman*, in the *Southern Journal of Homeopathy*, and in a reprint with which the state capitol was literally belittered. We quoted from memory at the time: now we have it before us; and there is no mistake about it; here it is, in the *Southern Journal of Homeopathy* for February, 1891, the official mouth-piece of the clan.

"But as to homeopathy, we maintain that the administration of homeopathic medicines does not jeopardize the lives of the community, and therefore does not come within the purview of restrictive legislation;" and further on, "why do they not leave us out altogether?" Is not this the same, substantially, as our quotation? To deny it is the shallowest subterfuge, as shallow as the claim itself, and as untrue. This assertion was made in a set address by the Hon. President of the homeopaths before the House Committee on Public Health on January 31, 1891, in session, where Drs. Bennett, Pope, Preston and Daniel were present, as also the homeopathic and electric delegation. We believe, the author does make the distinction of saying that the above sentiment was not uttered *in his office*, when the State Association Committee called on him. It matters not where he said it, it is a matter of record, and cannot be denied. Does not this look like an attempt to deceive? Did he once intimate to the committee that he and his disciples resort to the hypodermic syringe and morphine and atropine, and cocaine, and quinine, and chloral and strychnine, whenever they find their "potentized moonshine" unavailing, that is, as a rule, and in all grave cases? Not a bit of it! They said the administration of homeopathic medicine does not endanger life. This looks as if they would have the hearers believe they give homeopathic medicines—and nothing else! Or, does the gentleman wish us to think that he and his kind practice homeopathy solely, when we *know better?* or, is it that being a homeopath (?) morphine in his hands in ¼ gr. doses becomes a "homeopathic medicine" and *ergo*, is "harmless?"

"What then is the inevitable conclusion with reference to publicly declared homeopathists who openly confess that they do not confine themselves to homeopathy but practice all systems varying their methods in accordance with the whims of their patrons? The only possible conclusion is that such men are imposters! There is no principle in their professional services, and they are physicians for revenue only. They are ready to resort to any trick or device likely to secure them victims. Their sectarian title makes them quacks. Had homeopathy been obliged to rely upon the merits of its own particular achievements and not been habitually filching from legitimate medicine, no part of the earth would at this date be cursed with a homeopathist.''*

As stated in our last, the State Medical Association Committee taking them at their word—pretending, out of respect to the honorable gentlemen to believe that they *meant* it, introduced a clause in the bill now pending in the Senate, exempting from examination all those who confine their practice to the law of *similia similibus curantur* and infinitesimal doses. The homeopaths saying: "If you do this, it no longer concerns us; our mouths are necessarily closed"; but, *mirabile dictu!* since this was done they open their mouths still wider, and the wolf's ears stick out from the lamb's skin even more conspicuously than before! They now cry out for a "separate board," or equal representation on the one board! The clause exempts all honest homeopaths—if there be any; the spurious article, or those who under homeopathic guise practice what they call "allopathy" have no claim to exemption. Since this clause exempts what they claim to be and pretend to be—homeopaths—what have these persons to do with this or any other bill to regulate the practice of *medicine?* They should take their choice, stick to homeopathy, or, if they insist on practicing *medicine*, stand examination like a man.

But homeopaths (we do not mean the mixed breed, they represent still less), represent nothing; and in Texas especially represent nothing worthy of consideration. Numbering less than one hundred, or about 2% of the entire medical profession of the State, if counted as practitioners, should they be permitted—and especially by specious pleas and misrepresentations—to be a factor in medical legislation, to become a controlling element in the making of laws to govern the practice of *medicine?* They are very active at the Capitol; while, from the force of circumstances, the illness of Dr. Cupples, etc., the State Medical Associaciation bill has been left to take care of itself pretty much.

We could perhaps laugh at their arrogance were it not that they persist in impuging the motives of the medical profession in seeking legislation. Notwithstanding the bill is backed by a popular petition bearing 15,000 signatures; notwithstanding every State Medical Association in America has sought and secured, or is seeking such legislation, these busy-bodies, who, as shown, are neither one thing nor another, insist, and assert to intelligent legislators that it is a "plot" to crush them! To make such an assertion is to denounce the 60,000 practitioners of rational medicine in the United States as a set of scoundrels, banded together for a dishonorable purpose. It is contemptible! It is monstrous!

The Legislature cannot be blind to the cloven foot when they hear such things charged; for they know, personally, the venerable and honorable Dr. Cupples, a graduate of Edinburgh and of Paris; they know the venerable and honorable Dr. T. C. Osborn, the courtly and high-minded Paine, and Fly, and Swearingen; the scholarly Burroughs and Clopton; they know that no abler nor more honorable men ever lived than the members of the medical profession who are engaged in, or in sympathy with this needed reform—this effort to suppress quackery.

It is downright impertinence to intimate to an intelligent person that such men would lend themselves to any "plot" or "scheme" to "get control of the practice in Texas." Pray, tell us, somebody, what would these gentlemen be profited even by the total extinction of homeopathy? Let us see: There are some 300 counties in Texas, and 100 homeopaths all told. That is about ⅓ of an homeopath to each county. Now, suppose this Legislature could wipe out the "whole compoodle" at one stroke of the pen; let us see where we would be. This ⅓ of a man's practice would be divided, we will say, equally between the (estimated average of) fifteen doctors in the county. What would be each man's *pro-rata* share, supposing every homeopath in Texas to have a cash practice of $2000 a year—a big estimate? We have $2000 \div \frac{1}{3} = 666.66\frac{2}{3} \div 15 = \$44.44$ a year, or about $3.70 a month to each "regular"; a big thing; *quite* an inducement to sell one's honor. For very shame! But it is on a par with sailing under homeopathic colors and prescribing morphine; dodging behind an empty and meaningless title to escape examination, on a pretext that "homeopathic remedies are harmless."

But, *is* the administration of homeopathic remedies pure and simple, harmless? Let us see: *"Some of them [homeopathic remedies], being derived from the secretions from infectious diseases, cannot be deemed destitnte of danger. * * In 1886

so great was the demand for these remedies [?] that Dr. Samuel Swan found it profitable to issue a price list of them. These offered for sale, at a fixed price, on page 20, potentized "*pediculi pubis*" [crab-lice] "*pediculi capitis*"; "*pediculi corporis*"; page 1 offered "*acaris scabies*" [itch insect]. "Carbuncle of the neck, very severe" is offered as a remedy on page 6. Page 15 offers the "tears of a young girl in great suffering," under the name of "*lachryma filia*." On page 4 "*buboin*" or "*pus*" from a "*syphilitic bubo*" [!] Page 7 offers a preparation from "*chancre of syphlis*" [!]. Page 8 offers "colestrum" [the first milk of mother after birth of baby]. Page 9 "crotalide" [rattle-snake venom]. Cancer of the womb, bowels, breast, etc., are offered as remedies, as well as "*hippozinin*" from glanders. Osteo-necrosis [dead bone] pus, from a rectal abscess," etc., etc., ad nauseum.

"But to cap the climax of absurdity, in the same "price list" "rubrum," "flava," and "ceruli iridis"—or red, yellow and blue rays of the spectrum, as well as "Luna," or potentized [?] *moonshine!* are also tendered as therapeutic aids to the Hahnemaniac.' "*

[This is not jest, but dead serious easnest. See *Medical Standard* of February, 1891.—ED.]

No wonder "homeopaths" do not stick to their trade. What would some of their swell patrons, say to taking a dose of *body lice* or a little *pus* from a suppurating bubo from the person of a negro perhaps, or a Mexican? Ugh!

If anything can be more absurd than the serious proposal to use *moonshine* as a remedy(?) it is their boast to be able to render inert substances, like charcoal for instance, so powerful, by agitation or shaking, (and it must be done in a prescribed manner—"downward, always, and never in the opposite direction"—that a "dose," diluted to the *decillionth* degree would be '*too strong*, and a smell of the vial is prescribed."* Hahnemann says (in his *Materia Medica Pura*,") "if the patient is very sensative it will be sufficient for him to *smell once* of a vial containing a globule of the dilution." "They caution against *too many shakes* lest the medicine be thus rendered *too* violent."*

In the *Southern Journal of Homeopathy*, for February, 1891, edited by the arch-disciple of modified homeopathy, C. E. Fisher now of San Antonio, on page 421, in an article on crying babies we find the following: "Cries as if from being frightened by seeing Indians—*Stramonium.*"

That is to say to give a baby not crying, a *smell* of stramonium, would cause him to see "injuns;" beautiful theory!—

But enough. Will an intelligent legislature be hoodwinked by the claims of one hundred men who hold—or what is worse, pretend to hold such views as rational,—and are guilty of such shameless subterfuges; who practice or pretend to practice such stuff in the scared name of "medicine?" Then, in the name of consistency and of justice, make provision for, and recognize as a rational practice, the "Christian Science" abomination. It is much more respectable—because consistent; and surely it requires no stretch of imagination to believe as well in the efficacy of prayer as a therapeutic measure—as in the efficacy of moonshine and spectral rays,—or that the tears of "a young girl in great distress" will cure another girl in great distress; or if given to a happy girl will throw her into a state of "great distress." It is really too absurd for serious consideration; and yet—and yet—through the boldness and peristence of their pretensions many sensible and honest men are made to believe that these people represent a "school of medicine"—and that they are persecuted as rivals, by the practitioners of rational medicine. That clause in the constitution which says, "no preference shall ever be given to any school of medicine," so often quoted by the Hahnemaniacs, is proof of what we say, and is a great absurdity within itself.—*Vive la bagatelle.*

